# Supplementary material for: Integrative analysis reveals generalizable human neurodegenerative disease-associated glial states
Source: bioRxiv. 2025 Sep 25:2025.09.25.678630. Preprint. [Version 1] doi: 10.1101/2025.09.25.678630 (PMC12485690; doi:10.1101/2025.09.25.678630)
Supplement: 14 [file NIHPP2025.09.25.678630v1-supplement-14.pdf]

bioRxiv preprint doi: https://doi.org/10.1101/2025.09.25.678630; this version posted September 25, 2025. The copyright holder for this preprint (which was not certified by peer review) is the author/funder. This article is a US Government work. It is not subject to copyright under 17 USC 105 and is also made available for use under a CC0 license.

Supplementary Information

Supplementary figures include comparisons of glial taxonomy to published signatures and astrocyte subclustering (Figs. S1-2). Initial characterizations and disease associations are supported by proportion analysis, meta-analysis with *mashr*, cluster markers, and detailed cell counts (Fig. S3; Tables S1-13). An overview of pseudobulked differential expression, covariate selection, meta-analysis with *mashr*, and pathway analysis are available online (Figs S4-8; Tables 14-28). To define the hnDAM signature information includes latent variable discovery, pathway analysis, transcription factor analysis, validation in a replication cohort, and transcriptional programming discovery (Figs S9-10, Tables S29-40). Finally, results pertaining to *in vitro* perturbation of iPSC-derived microglia are also available online (Tables S41-43). A stable release of the supplementary information associated with this pre-print can be found on Zenodo (10.5281/zenodo.17203361).

Fig. S1. Cross-NDD glial substate diversity reflected in published human and mouse signatures. GSEA-style enrichment performed in *fgsea* against published disease-associated glial subtypes (see **"Characterization of cell subtypes"**). (**A**) Total and (**B**) cortical astrocyte annotations segregated mainly by fibrous (*GFAP*+) and protoplasmic (*GFAP*-) recapitulate 26 known astrocytic substates from three human studies. (**C**) Unified naming strategy captures the transcriptional state of 31 microglial substates across seven human and two mouse studies. (**D**) *De novo* oligodendrocyte clusters recapitulates limited known heterogeneity of 17 oligodendrocytic sub-states from two human studies.

Fig. S2. Astrocyte transcriptional state driven by regional heterogeneity. (A) Cortical astrocytes (HIP, EC, AnG, M1, FC/PFC, OC/V1, TC/MTG) from initial clustering (Fig. 1A). (**B**) Successive subclustering reveals 6 clusters at a resolution of 0.15. (**C**) Allocortical (HIP/EC) and neocortical regions (AnG, M1, FC/PFC, OC/V1, TC/MTG) display minimal sub-regional heterogeneity. (**D**) Subcortical astrocytes (DMV, GPi, TH) from initial clustering (Fig. 1A). (**E**) Successive sub-clustering reveals 9 clusters at a resolution of 0.2. (**F**) Substantial sub-regional heterogeneity apparent across 3 subcortical regions.

Fig. S3. Astrocyte and oligodendrocyte subpopulations lack strong cross-NDD associations. Differential abundance of subtype populations was tested using a GLM covaried by sex and age and β and p-values were refined using *mashr*[73]. (**A**) Astrocytic differential abundance reveals 2 disease-associated subtypes across NDD and regions (* LFSR < *0.05*) (**B**) Per sample proportion changes of DAA-like subtypes in AD, C9TLD, FTD-GRN (CITE). (**C**) Oligodendrocytic differential abundance reveals 1

bioRxiv preprint doi: https://doi.org/10.1101/2025.09.25.678630; this version posted September 25, 2025. The copyright holder for this preprint (which was not certified by peer review) is the author/funder. This article is a US Government work. It is not subject to copyright under 17 USC 105 and is also made available for use under a CC0 license.

disease-associated subtypes across NDD and regions (**** LFSR < *0.0001*) (**D**) Per sample proportion changes of DAO-like subtypes in AD, C9TLD, PD (CITE). See **Table S13** for summary statistics.

Fig. S4. Overview of differential expression workflow. (**A**) Feature and sample filtering strategies for pseudo-bulk and cell-level differential expression. Latent factors to be included as covariates were selected automatically using *RUVseq* (See **"Differential gene expression analysis"**). (**B**) Example of automated hyperparameter selection to define components of unwanted variance.

Fig. S5. Summary of pseudobulk and cell-level differential expression results. (**A-C**) Number of differentially expressed genes at the summarized, number of cells past QC filters, number of donors contributing cells, and median UMIs per condition [disease x region] across respective glial types. (**D**) A histogram of spearman correlation of *DESeq2* and *nebula* results per condition [disease x region]. (**E**) Correlation of z-scored log2FC for select comparison reveals correlation coefficient equal to one (Spearman ρ = 1).

Fig. S6. Cross-disease meta-analysis of microglial gene expression reveals unique association with cognitive resilience. (**A**) Mixture proportions of different types of covariance matrices generated from *mashr* analysis of microglia reveals data-driven model (ED_tPCA) explains the most sharing of cell-level differential expression profiles (computed by *nebula*). (**B**) Covariance matrix demonstrates patterns of shared variance explained by ED_tPCA. (**C**) K-means clustering (k = 6) of genes with sharing most explained by ED_tPCA (n = 2,500). GO terms computed in *ClusterProfiler* reveal immune-processes in the union of clusters one and two (**D**) and no significantly enriched pathways in the union of clusters four and six (**E**).

Fig. S7. Cross-disease meta-analysis of astrocyte gene expression reveals associations with ALS and cognitive resilience. (**A**) Mixture proportions of different types of covariance matrices generated from *mashr* analysis of astrocytes reveals data-driven model (ED_tPCA) explains the most sharing of cell-level differential expression profiles (computed by *nebula*). (**B**) Covariance matrix demonstrates patterns of shared variance explained by ED_tPCA. (**C**) K-means clustering (k = 6) of genes with sharing most explained by ED_tPCA (n = 3,502). GO terms computed in *ClusterProfiler* reveal aerobic respiration in cluster two (**D**) and immune-related pathways in cluster five (**E**).

Fig. S8. Cross-disease meta-analysis of oligodendrocyte gene expression reveals associations with ALS. (**A**) Mixture proportions of different types of covariance

bioRxiv preprint doi: https://doi.org/10.1101/2025.09.25.678630; this version posted September 25, 2025. The copyright holder for this preprint (which was not certified by peer review) is the author/funder. This article is a US Government work. It is not subject to copyright under 17 USC 105 and is also made available for use under a CC0 license.

matrices generated from *mashr* analysis of oligodendrocytes reveals data-driven model (ED_tPCA) explains the most sharing of cell-level differential expression profiles (computed by *nebula*). (**B**) Covariance matrix demonstrates patterns of shared variance explained by ED_tPCA. (**C**) K-means clustering (k = 5) of genes with sharing most explained by ED_tPCA (n = 1,238). GO terms computed in *ClusterProfiler* reveal aerobic respiration in cluster two (**D**) and membrane trafficking-related pathways in cluster three (**E**).

Fig. S9. Latent factorization of microglial gene expression programs. The definition of the hnDAM module was contingent on the discovery of latent factors shared across disease-associated subtypes. Consensus non-negative matrix factorization allowed for the discovery of factors within each study of the discovery cohort (See **Methods**). The number of gene expression programs (GEPs) were determined: (**A**) 16 GEPs for Gerrits_2022, (**B**) 12 GEPs for Mathys_2024, (**C**) 12 GEPs for Pineda_2024, (**D**) 17 GEPs for NM_2024. Heatmaps of scaled GEP usage across microglia substates represented as heatmaps.

Fig. S10. Cross-study identification of a border-associated macrophage gene expression program. Microglial gene expression programs were defined using *cNMF*[83] (see "**Microglia signature discovery via latent factorization**"). (**A**) GEPs uniquely enriched in border-associated macrophages were apparent from all four discovery studies. (**B**) Genes were prioritized from Gerrits_2022_GEP9, Mathys_2024_GEP6, Pineda_2024_GEP6, and NM_2024_GEP4 by z-scoring eigenvalues within respective GEPs. Z-scores for each gene were averaged across study to define the BAM signature (149 genes, average z-score > 5). (**C-D**) Expression of BAM genes across microglial subtypes shows enrichment in one BAM cluster. A module score was assigned to each cell using the *AddModuleScore()* function (**C**), or expression was computed using the *AverageExpression()* function in *Seurat*, and z-scored across subtypes (**D**). (**E**) Comparison of BAM signature to hnDAM (**Fig. 3**) highlights discrete gene membership. (**F**) hnDAM genes are neither reversed (negative value), nor similarly enriched (positive value) when ranked by cross-study z-score of BAM signature.

bioRxiv preprint doi: https://doi.org/10.1101/2025.09.25.678630; this version posted September 25, 2025. The copyright holder for this preprint (which was not certified by peer review) is the author/funder. This article is a US Government work. It is not subject to copyright under 17 USC 105 and is also made available for use under a CC0 license.

Supplemental Data

AMP-PD: Data used in the preparation of this article were obtained from the Accelerating Medicine Partnership® (AMP®) Parkinson's Disease (AMP PD) Knowledge Platform. For up-to-date information on the study, visit https://www.amp-pd.org.

The AMP® PD program is a public-private partnership managed by the Foundation for the National Institutes of Health and funded by the National Institute of Neurological Disorders and Stroke (NINDS) in partnership with the Aligning Science Across Parkinson's (ASAP) initiative; Celgene Corporation, a subsidiary of Bristol-Myers Squibb Company; GlaxoSmithKline plc (GSK); The Michael J. Fox Foundation for Parkinson's Research ; Pfizer Inc.; AbbVie Inc.; Sanofi US Services Inc.; and Verily Life Sciences. ACCELERATING MEDICINES PARTNERSHIP and AMP are registered service marks of the U.S. Department of Health and Human Services.

AD Knowledge Portal: The results published here are in whole or in part based on data obtained from the AD Knowledge Portal (https://adknowledgeportal.org/).

The MIT ROSMAP Single-Nucleus Multiomics Study: Study data were generated from postmortem brain tissue provided by the Religious Orders Study and Rush Memory and Aging Project (ROSMAP) cohort at Rush Alzheimer's Disease Center, Rush University Medical Center, Chicago. This work was supported in part by the Cure Alzheimer's Fund, NIH grants AG058002, AG062377, NS110453, NS115064, AG062335, AG074003, NS127187, MH119509, HG008155 (M.K.), RF1AG062377, RF1 AG054321, RO1 AG054012 (L.-H.T.) and the NIH training grant GM087237 (to C.A.B.). ROSMAP is supported by P30AG10161, P30AG72975, R01AG15819, R01AG17917. U01AG46152, U01AG61356.

ROSMAP: Study data were generated from postmortem brain tissue provided by the Religious Orders Study and Rush Memory and Aging Project (ROSMAP) cohort at Rush Alzheimer's Disease Center, Rush University Medical Center, Chicago. This work was funded by NIH grants U01AG061356 (De Jager/Bennett), RF1AG057473 (De Jager/Bennett), and U01AG046152 (De Jager/Bennett) as part of the AMP-AD consortium, as well as NIH grants R01AG066831 (Menon) and U01AG072572 (De Jager/St George-Hyslop).

Seattle-AD: Study data were generated from postmortem brain tissue obtained from the University of Washington BioRepository and Integrated Neuropathology (BRaIN) laboratory and Precision Neuropathology Core, which is supported by the NIH grants for the UW Alzheimer's Disease Research Center (P50AG005136 and P30AG066509) and

bioRxiv preprint doi: https://doi.org/10.1101/2025.09.25.678630; this version posted September 25, 2025. The copyright holder for this preprint (which was not certified by peer review) is the author/funder. This article is a US Government work. It is not subject to copyright under 17 USC 105 and is also made available for use under a CC0 license.

the Adult Changes in Thought Study (U01AG006781 and U19AG066567). This study is supported by NIA grant U19AG060909.

PPMI (Parkinson's Progression Markers Initiative) Biospecimen Use Agreement: Data biospecimens used in the analyses presented in this article were obtained from the Parkinson's Progression Markers Initiative (PPMI) (www.ppmi-info.org/access-dataspecimens/download-data). As such, the investigators within PPMI contributed to the design and implementation of PPMI and/or provided data and collected biospecimens, but did not participate in the analysis or writing of this report. For up-to- date information on the study, visit www.ppmi-info.org. PPMI – a public-private partnership – is funded by The Michael J. Fox Foundation for Parkinson's Research and funding partners. A full list of PPMI funding partners can be found under the "who we are" tab on the PPMI website (https://www.ppmi-info.org/about-ppmi/who-we-are/study-sponsors).

bioRxiv preprint doi: https://doi.org/10.1101/2025.09.25.678630; this version posted September 25, 2025. The copyright holder for this preprint (which was not certified by peer review) is the author/funder. This article is a US Government work. It is not subject to copyright under 17 USC 105 and is also made available for use under a CC0 license.

Fig. S1

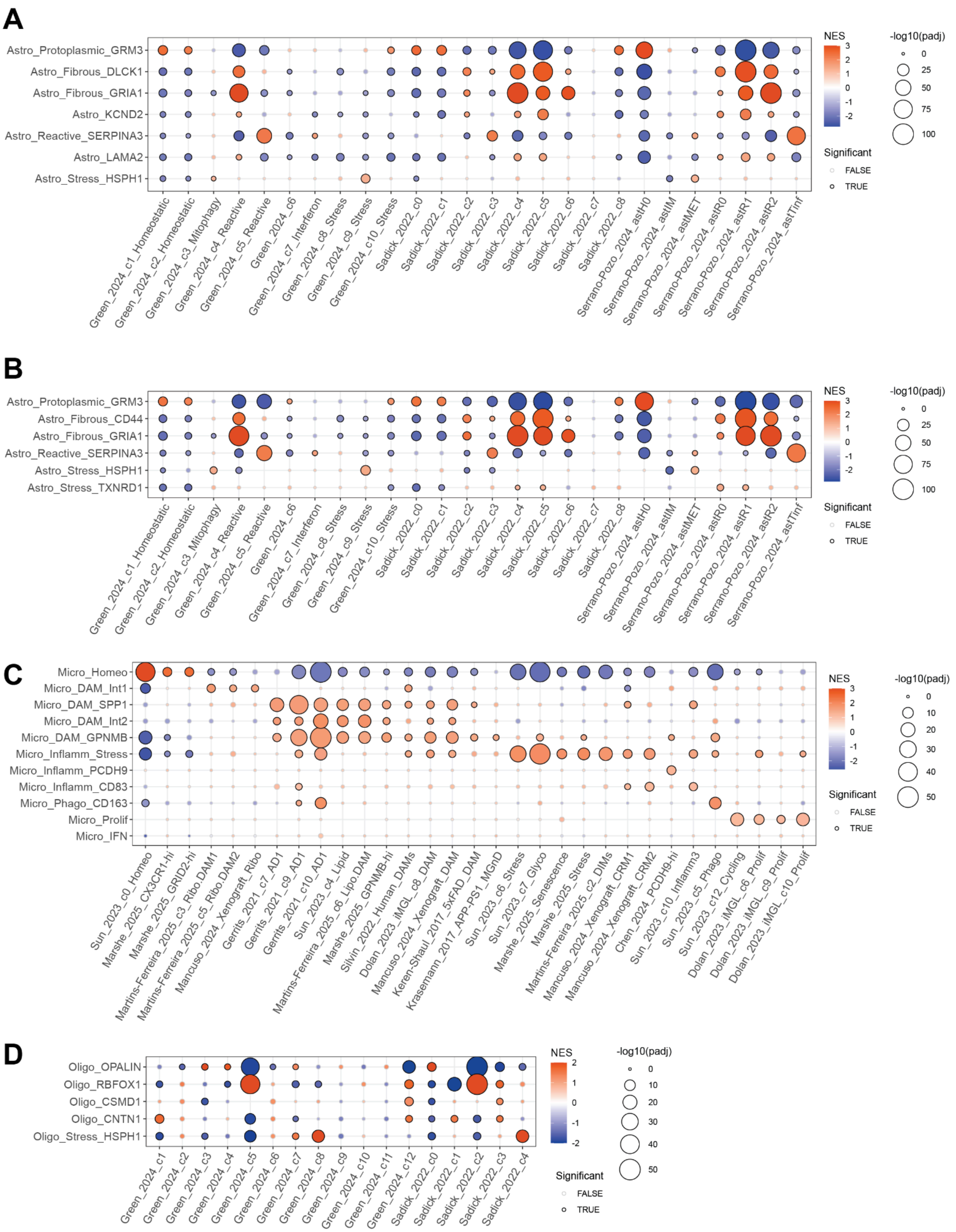

Fig. S1

bioRxiv preprint doi: https://doi.org/10.1101/2025.09.25.678630; this version posted September 25, 2025. The copyright holder for this preprint (which was not certified by peer review) is the author/funder. This article is a US Government work. It is not subject to copyright under 17 USC 105 and is also made available for use under a CC0 license.

Fig. S2

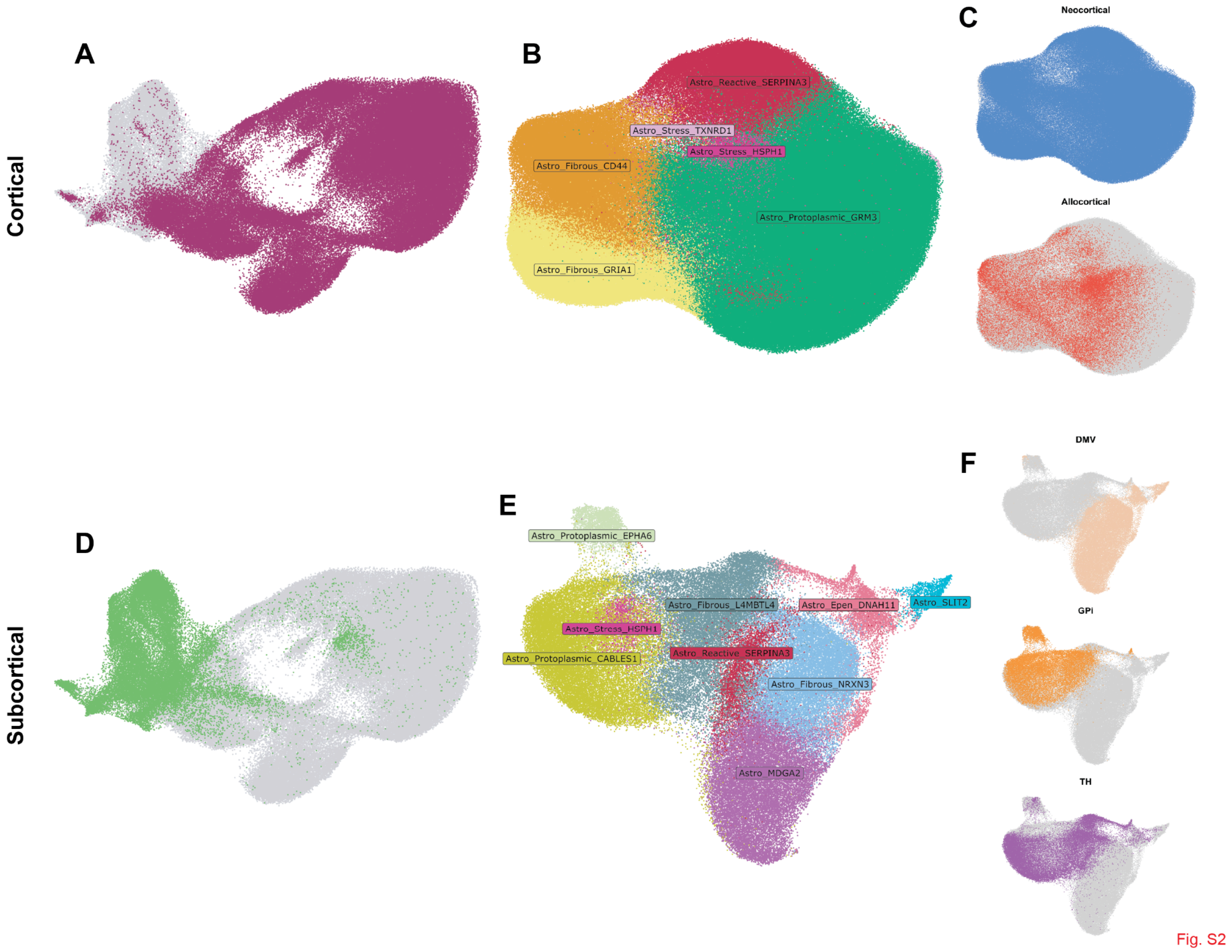

Fig. S2

bioRxiv preprint doi: https://doi.org/10.1101/2025.09.25.678630; this version posted September 25, 2025. The copyright holder for this preprint (which was not certified by peer review) is the author/funder. This article is a US Government work. It is not subject to copyright under 17 USC 105 and is also made available for use under a CC0 license.

Fig. S3

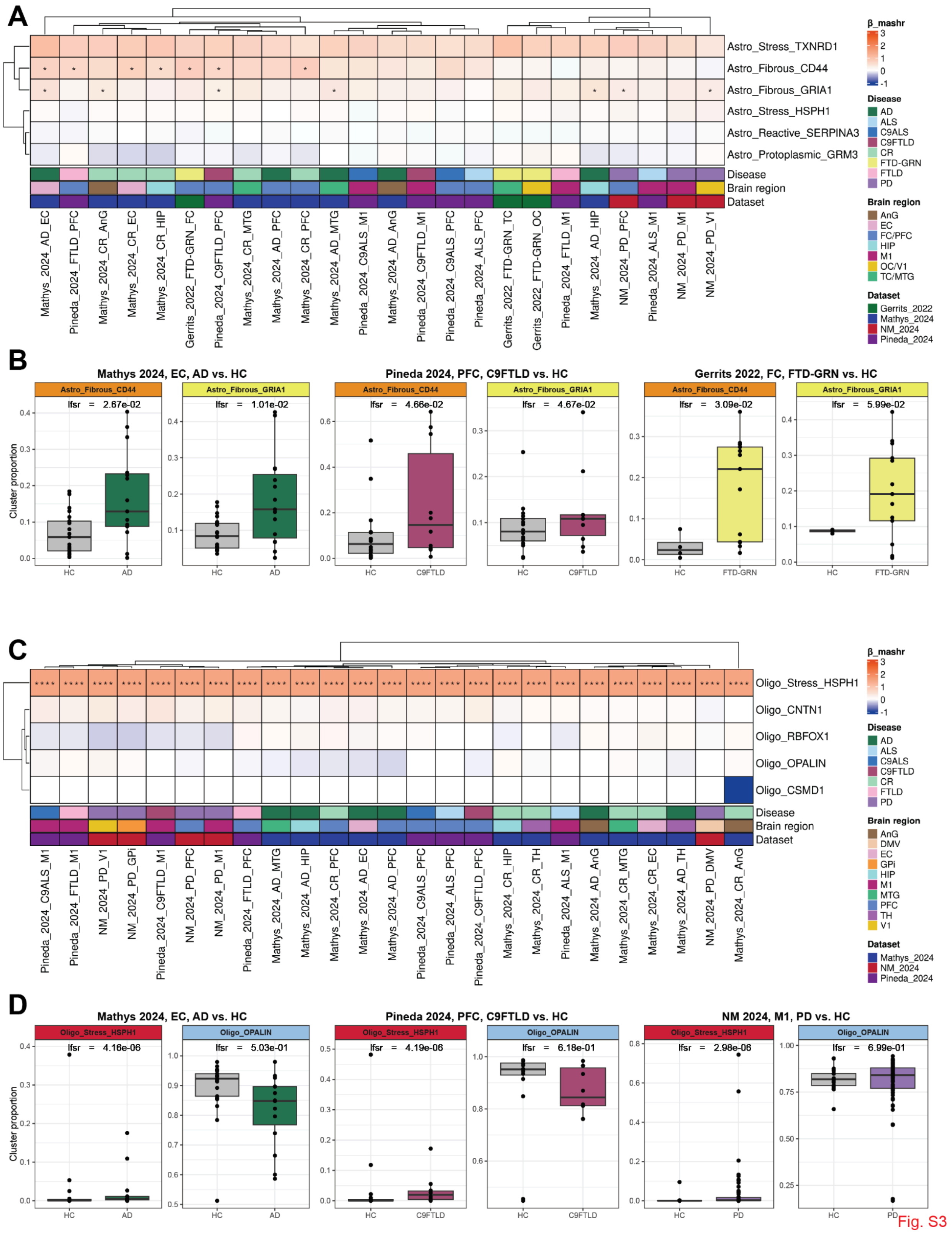

Fig. S3

bioRxiv preprint doi: https://doi.org/10.1101/2025.09.25.678630; this version posted September 25, 2025. The copyright holder for this preprint (which was not certified by peer review) is the author/funder. This article is a US Government work. It is not subject to copyright under 17 USC 105 and is also made available for use under a CC0 license.

Fig. S4

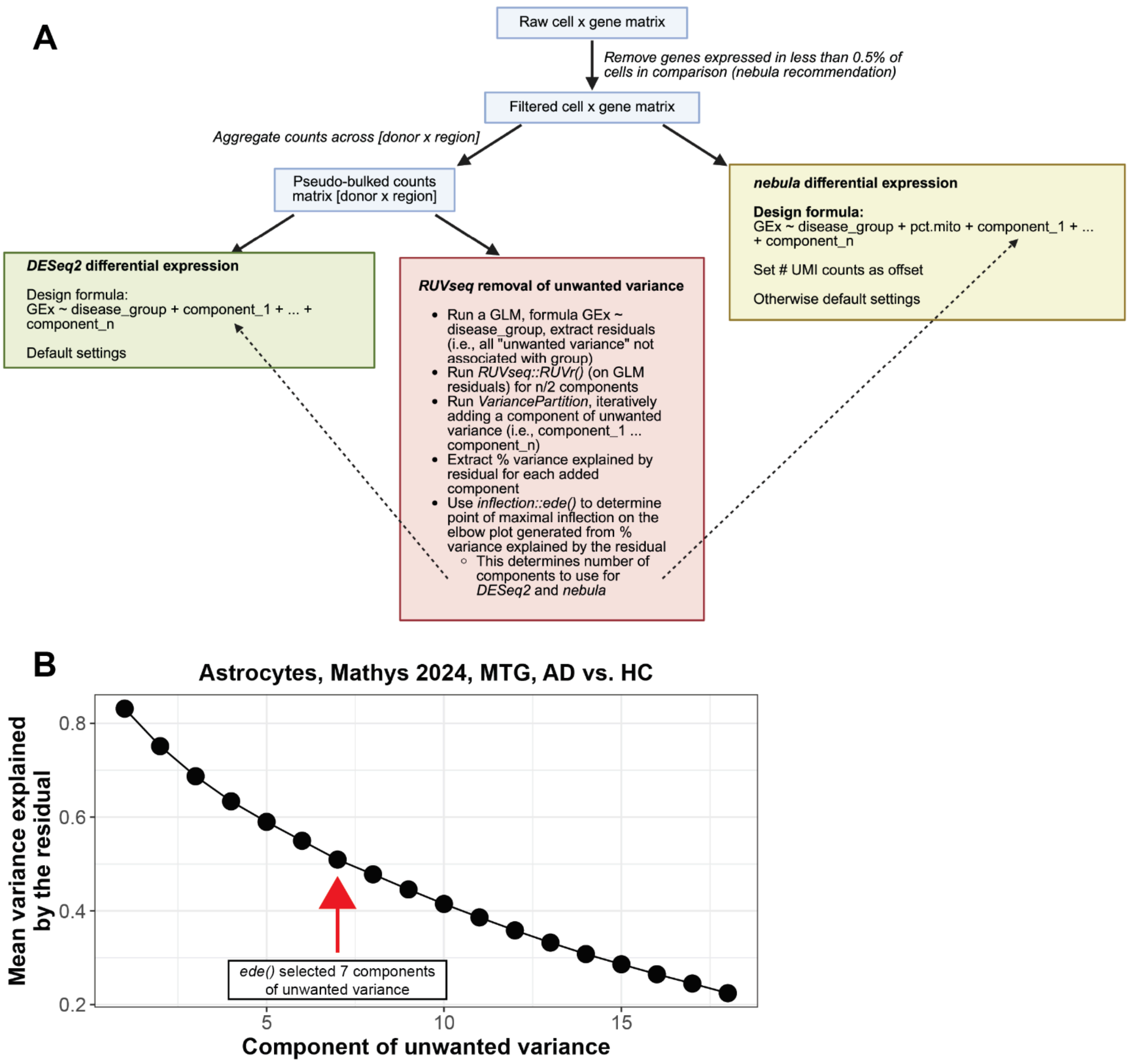

Final DESeq2 design formula: ~group + component_1 + ... + component_7
Final nebula design formula: ~group + pct.mito + component_1 + ... + component_7

bioRxiv preprint doi: https://doi.org/10.1101/2025.09.25.678630; this version posted September 25, 2025. The copyright holder for this preprint (which was not certified by peer review) is the author/funder. This article is a US Government work. It is not subject to copyright under 17 USC 105 and is also made available for use under a CC0 license.

Fig. S5

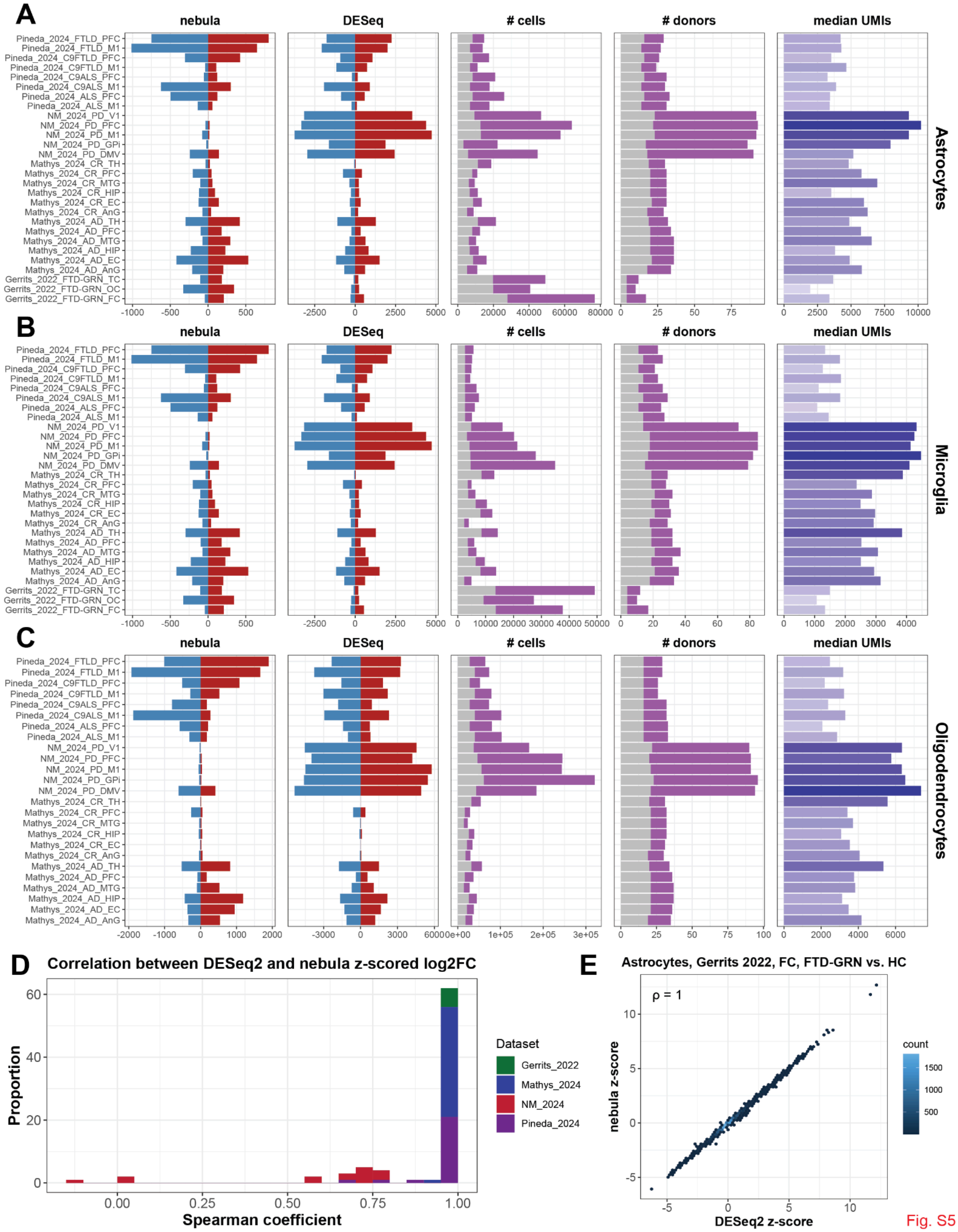

Fig. S5

bioRxiv preprint doi: https://doi.org/10.1101/2025.09.25.678630; this version posted September 25, 2025. The copyright holder for this preprint (which was not certified by peer review) is the author/funder. This article is a US Government work. It is not subject to copyright under 17 USC 105 and is also made available for use under a CC0 license.

Fig. S6

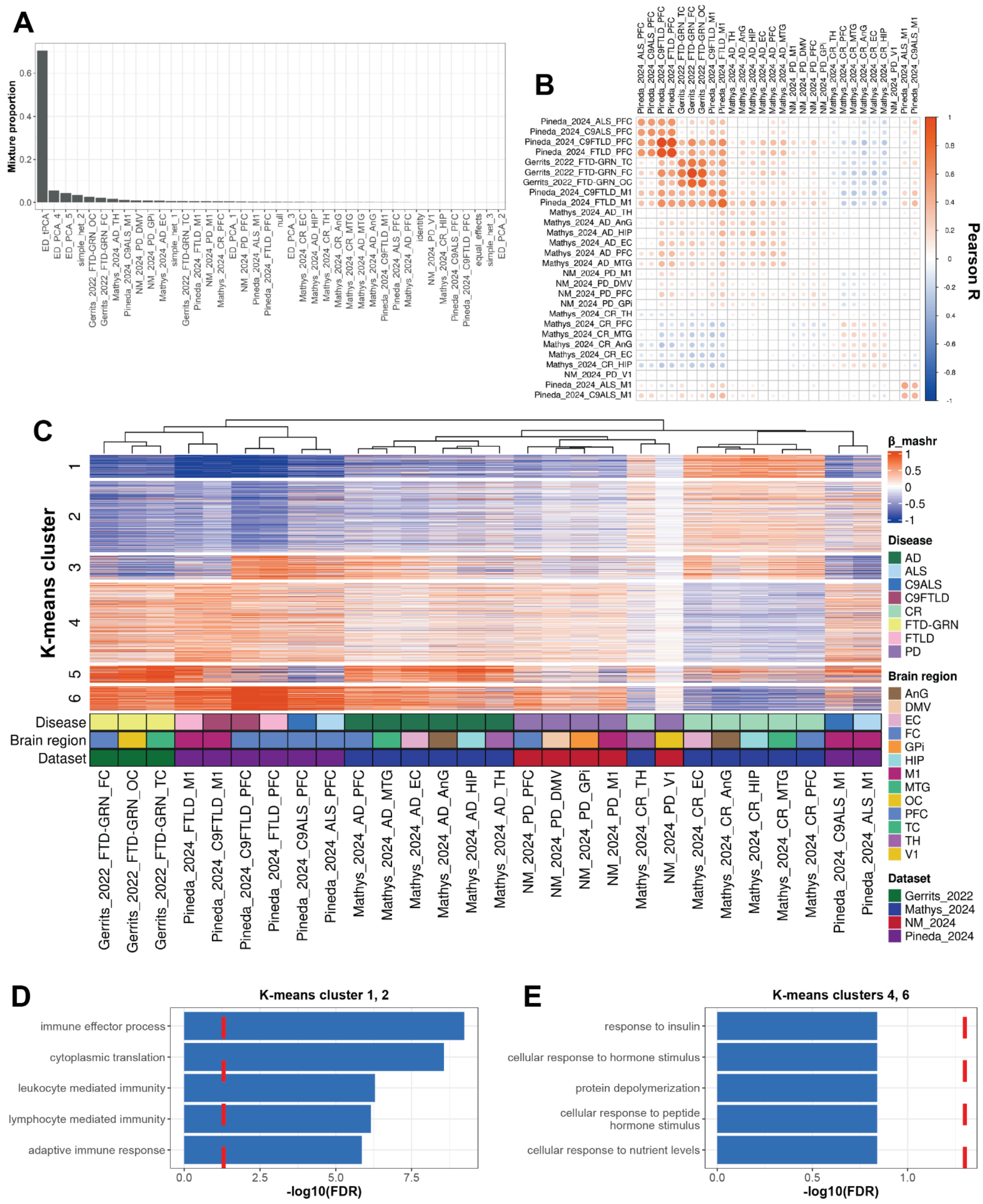

Fig. S6

bioRxiv preprint doi: https://doi.org/10.1101/2025.09.25.678630; this version posted September 25, 2025. The copyright holder for this preprint (which was not certified by peer review) is the author/funder. This article is a US Government work. It is not subject to copyright under 17 USC 105 and is also made available for use under a CC0 license.

Fig. S7

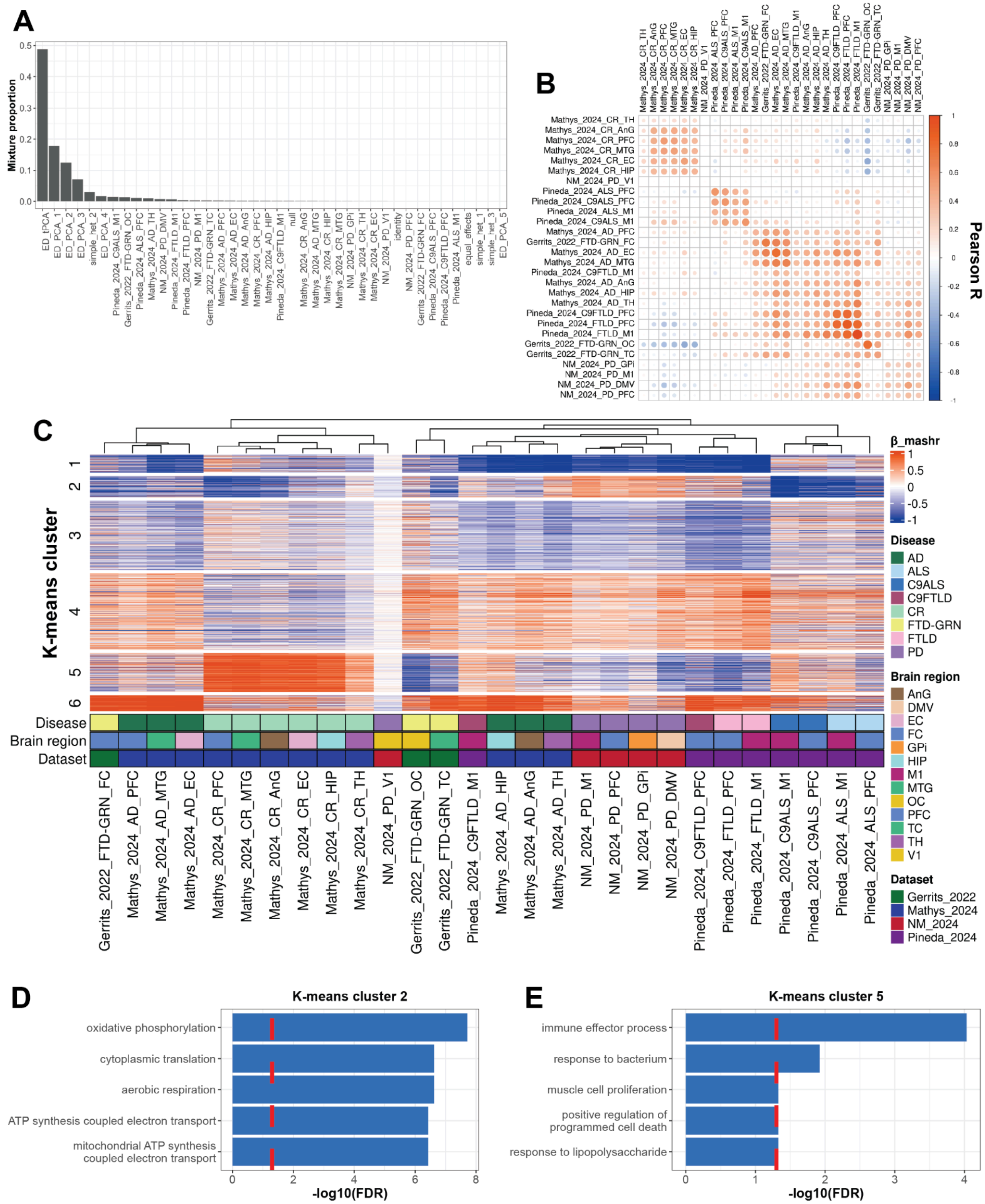

Fig. S7

bioRxiv preprint doi: https://doi.org/10.1101/2025.09.25.678630; this version posted September 25, 2025. The copyright holder for this preprint (which was not certified by peer review) is the author/funder. This article is a US Government work. It is not subject to copyright under 17 USC 105 and is also made available for use under a CC0 license.

Fig. S8

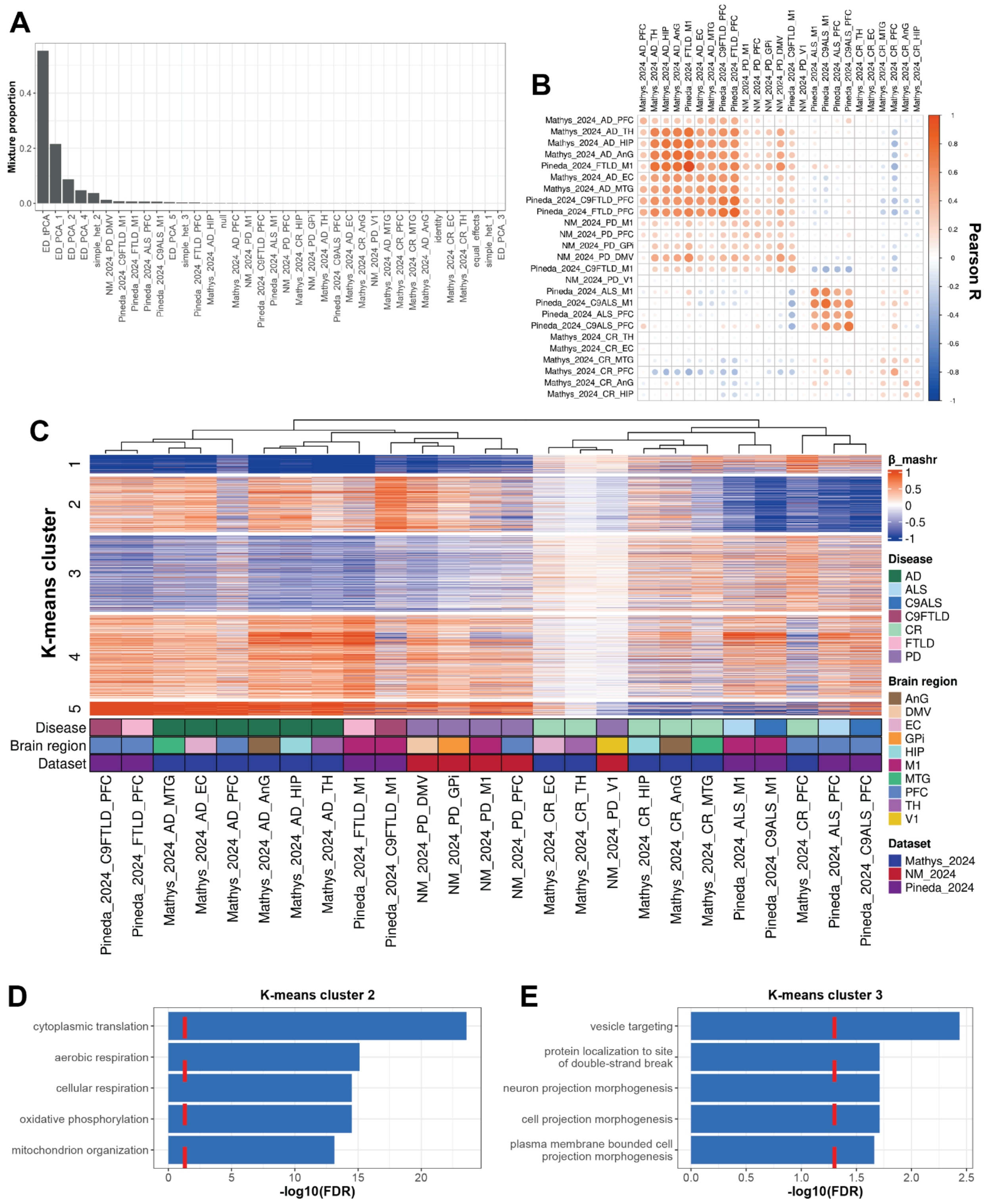

Fig. S8

bioRxiv preprint doi: https://doi.org/10.1101/2025.09.25.678630; this version posted September 25, 2025. The copyright holder for this preprint (which was not certified by peer review) is the author/funder. This article is a US Government work. It is not subject to copyright under 17 USC 105 and is also made available for use under a CC0 license.

Fig. S9

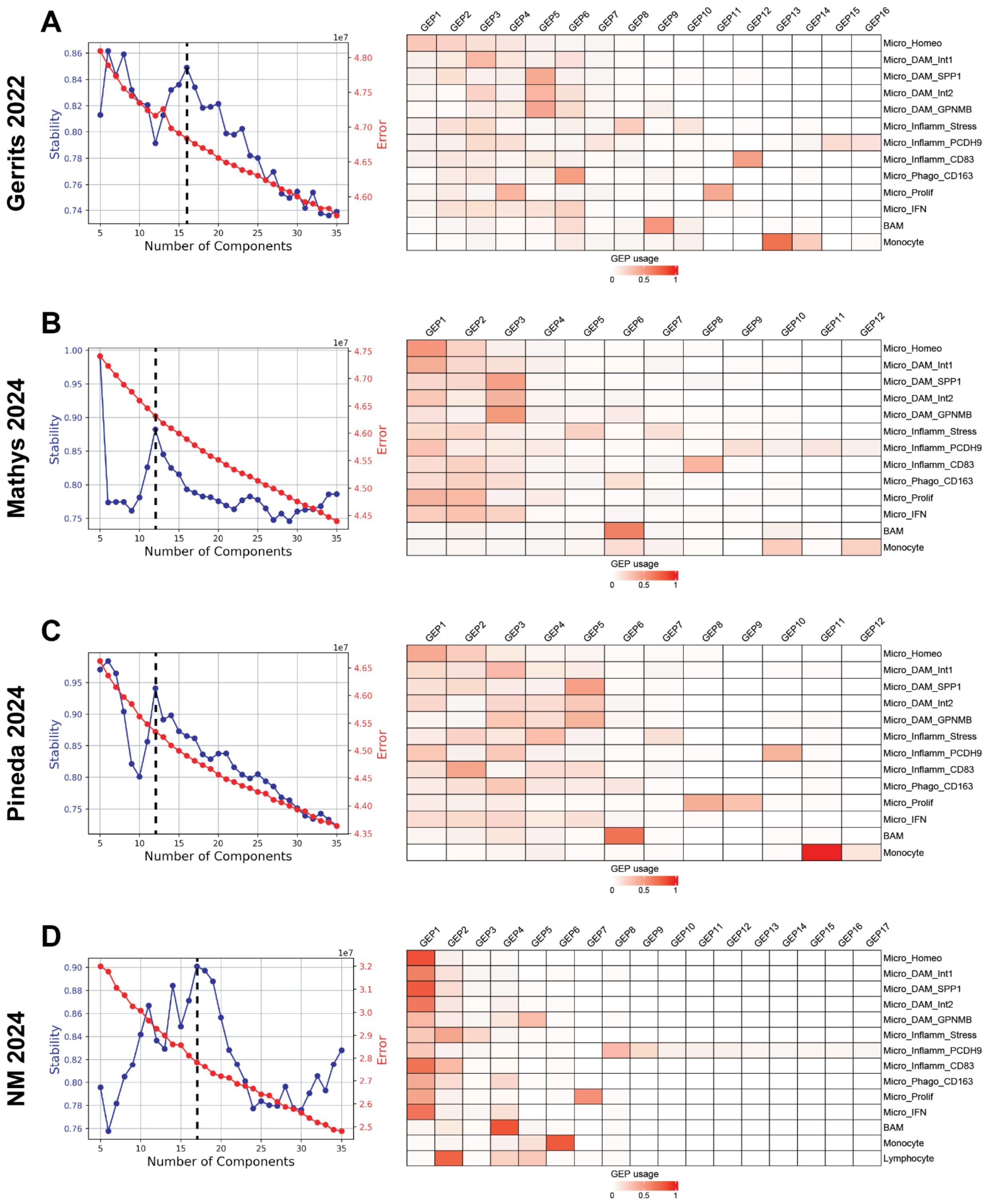

Fig. S9

bioRxiv preprint doi: https://doi.org/10.1101/2025.09.25.678630; this version posted September 25, 2025. The copyright holder for this preprint (which was not certified by peer review) is the author/funder. This article is a US Government work. It is not subject to copyright under 17 USC 105 and is also made available for use under a CC0 license.

Fig. S10

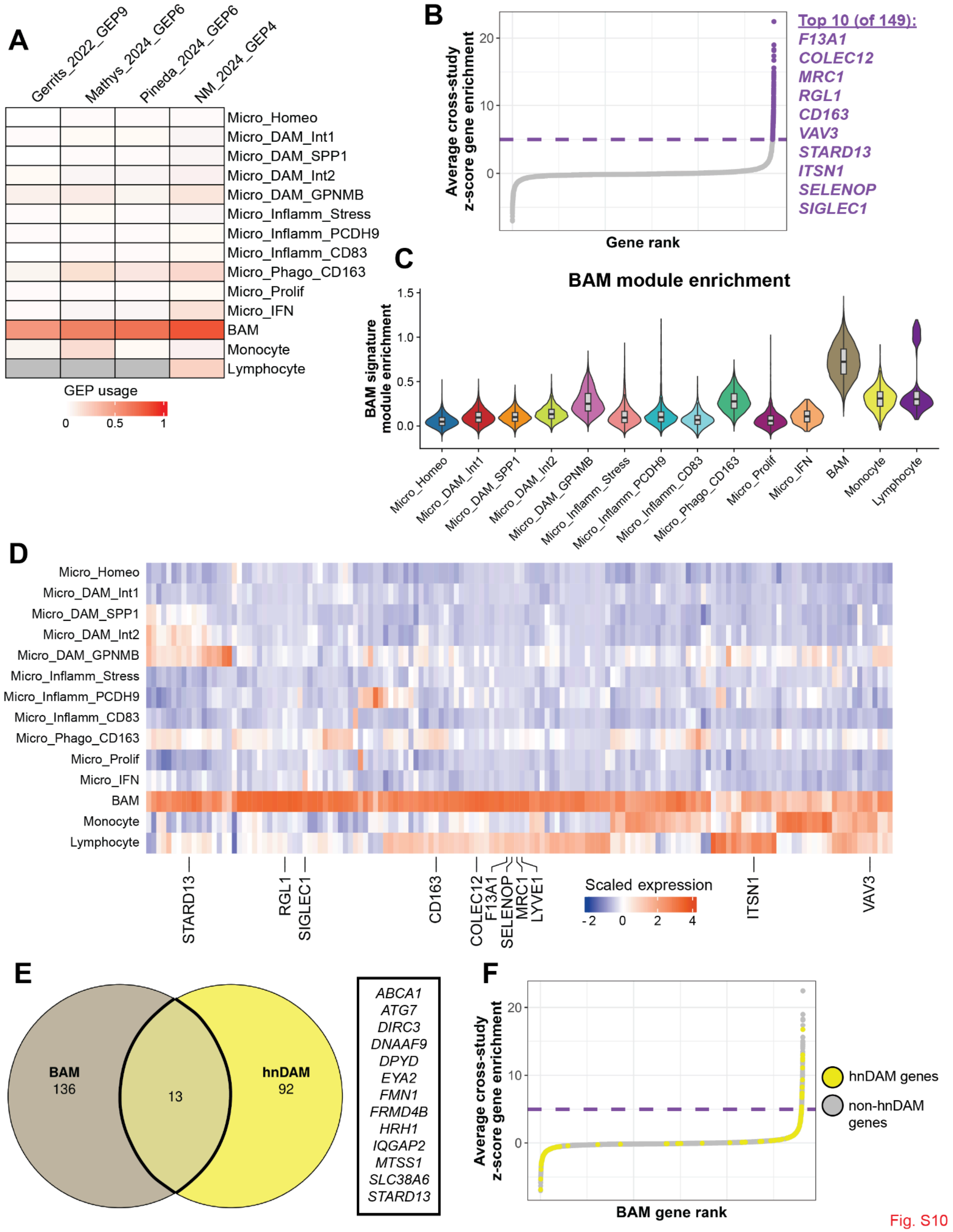

Fig. S10
